# 

**Published:** 1916-03-11

**Authors:** 


### CONTENTS.

PAGE
**The Pressure on the Profession** ... ... ... 515
**The Canonisation of Research** ... ... ... 516
**Hospital and Institutional News** ... ... ... 517
Lord Knutsford—The Late Captain Halford Burdett—Research in Military Surgery—What is a "National Necessity"?—A "Certain Liveliness" at Cardiff—Advertising for a Farm—A Secretary's "Salary" of £15 a Year—An Absolutely Original Appeal—The Growth of Oral Surgery—A Hospital Chairman's Unique Example—A Chairman on the Spot—The Extension of Ancoats Hospital—"Discharged for Drunkenness"—The Necessary Complement to Schools for Mothers—The Centre of Attraction in Hospital Work—King's College Hospital—The Prudential Assurance Company—Tropical Medicine and the Royal Southern Hospital, Liverpool—The Last Word in Fire Precautions—A Pioneer of After-Care—Consumptives in the Army: A Useful Suggestion—Provision for the Insane—East Sussex Tuberculosis Scheme—Margarine *versus* Butter—Contracts for Medical Supplies—Nurses Victims of False Economy—This Week's Drug Market.
**The Prevention of Venereal Diseases** ... ... 523
The Report of the Royal Commission.
**Parliament and the Profession** ... ... ... 524
The Advisory Medical Board—Territorial Base Hospitals—Hospital Accommodation for Wounded Soldiers—R.A.M.C. Prisoners in Germany—The Distribution of Medical Officers at the Front—Morphine for the Army—Other Matters of Interest.

PAGE

**Trench Fever** ... ... ... ... ... ... 525

A Diagnostic Dumping Ground?

**A Medical Causerie** ... ... ... ... ... 526

**A Word of Caution to Hospital Buyers** ... 527

Have Drugs Passed their Highest Price?

**Hours of Work for Women** ... ... ... 528

Night Work for Nurses and Munition Workers.

**The Matrons' and Sisters' Department** ... 529

A Nursing Mess and its Teaching.

Round the Hospitals.

A Medal for War Service—Jam at 2d. per lb.—Nurses after the War—A Ward Sister's Standards—Faith in our Workers.

**The School Doctor** ... ... ... ... ... 531

Exclusions.

**St. Marylebone Health Society** ... ... ... 532

**The Editor's Letter-Box** ... ... ... ... 533

Where a Nursing Sister was not even a Nurse—"War Wastage"—The Price of Gas.

**Annual Meetings** ... ... ... ... ... 534

**The Recruitment of Doctors in England and Wales** ... ... ... ... ... ... 534

**Passing Events and Topics** ... ... ... ... 534

**Institutional Needs** ... ... ... ... ... 534

**Gifts and Bequests** ... ... ... ... ... iv

**Medical Appointments** ... ... ... ... ... iv

**Obituary** ... ... ... ... ... ... ... iv

**The Book Market** ... ... ... ... ... iv

**The Institutional Worker** ... ... (Suppl.) 1

Economies in the Matron's Department: Answers to February Question.

